# Targeting tumor-associated macrophages in colon cancer: mechanisms and therapeutic strategies

**DOI:** 10.3389/fimmu.2025.1573917

**Published:** 2025-03-21

**Authors:** Jianqin Xiang, Jian Wang, Huihui Xiao, Chengchen Huang, Chunrong Wu, Lin Zhang, Chenyuan Qian, Debing Xiang

**Affiliations:** ^1^ Department of Oncology, The Affiliated Hospital of Southwest Medical University, Luzhou, Sichuan, China; ^2^ Department of Oncology, Chongqing University Jiangjin Hospital, Chongqing, China; ^3^ Department of Gastroenterology, Chongqing University Jiangjin Hospital, Chongqing, China

**Keywords:** colon cancer, tumor-associated macrophages, M1 macrophages, immune suppression, metastasis, chemokines

## Abstract

Colon cancer (CC) remains a primary contributor to cancer-related fatalities worldwide, driven by difficulties in early diagnosis and constrained therapeutic options. Recent studies underscore the importance of the tumor microenvironment (TME), notably tumor-associated macrophages (TAMs), in fostering malignancy progression and therapy resistance. Through their inherent plasticity, TAMs facilitate immunosuppression, angiogenic processes, metastatic spread, and drug tolerance. In contrast to M1 macrophages, which promote inflammatory and tumoricidal responses, M2 macrophages support tumor expansion and dissemination by exerting immunosuppressive and pro-angiogenic influences. Consequently, manipulating TAMs has emerged as a potential avenue to enhance treatment effectiveness. This review outlines the origins, polarization states, and functions of TAMs in CC, highlights their role in driving tumor advancement, and surveys ongoing efforts to target these cells for better patient outcomes. Emerging therapeutic strategies aimed at modulating TAM functions - including depletion strategies, reprogramming approaches that shift M2-polarized TAMs toward an M1 phenotype, and inhibition of key signaling pathways sustaining TAM-mediated immunosuppression-are currently under active investigation. These approaches hold promise in overcoming TAM - induced resistance and improving immunotherapeutic efficacy in CC.

## 1 Introduction

Colon cancer (CC), a frequent malignancy of the gastrointestinal tract, has outcomes and therapeutic responses shaped by various contributing factors (1). More than one million new diagnoses emerge across the globe annually, making it the second leading contributor to cancer-associated mortality (2). Limited survival rates largely stem from obstacles in early detection and a paucity of potent treatment strategies. Current protocols primarily center on surgery, complemented by radiation and chemotherapy. As overall health standards advance, there is a pressing demand for novel therapeutic avenues and refined prognostic tools (3–5). Recent findings underscore the importance of targeting the tumor microenvironment (TME) alongside conventional interventions to bolster treatment efficacy (6–9).

Established malignancies often present a suppressive microenvironment that impairs antitumor immunity and drives cancer progression (10, 11). Within these “immune evasion mechanisms,” tumor cells circumvent normal immune processes by modifying antigen presentation, releasing suppressive signals that promote lymphocyte apoptosis, or engaging detrimental regulatory pathways (12–14). These strategies evolve over the course of tumor development, growing ever more varied and complex. Consequently, disrupting these pathways and reactivating host immunity remain significant hurdles for tumor immunotherapy. The underlying factors behind tumor evasion are highly intricate, with the TME exerting a pivotal influence. Macrophages, which can comprise up to half of the tumor’s cellular mass, are the most abundant infiltrating immune cells in the TME (15). Termed tumor-associated macrophages (TAMs), they facilitate tumor establishment, metastatic spread, immune suppression, and vascularization (16–18).

In the setting of CC, TAMs play a central role in disease onset and progression, highlighting their potential as immunotherapeutic targets. Yet, their functions are multifaceted, and either depleting these macrophages or modifying their phenotypes may boost anti-tumor immunotherapy (19). This review synthesizes current findings on TAM origins, polarization states, and overall roles. It also examines how TAMs facilitate CC initiation and progression, underscores emerging TAM-focused strategies for personalized diagnostics and treatment, and outlines prospective challenges. These insights are intended to spur the development of TAM-oriented precision medicine in CC.

## 2 Origin and polarization of TAMs

Monocytes and macrophages circulating in the bloodstream represent the principal source of TAMs. Their entry into specific regions of the TME is governed by various cytokines, including C-C motif chemokine ligand 2/5/7 (CCL2/5/7), C-X-C motif chemokine ligand 8/12 (CXCL8/12), vascular endothelial growth factor (VEGF), and transforming growth factor-β (TGF-β) (20). Macrophages exhibit notable plasticity, transitioning into M1 or M2 phenotypes depending on local cues. M1 TAMs primarily initiate Th1 responses, amplify inflammation, and exert tumoricidal activity (21, 22). M2 TAMs secrete VEGF, matrix metalloproteinases (MMPs), and epidermal growth factor (EGF), displaying elevated IL-10 and reduced IL-12 levels. M2 TAMs predominantly engage in Th2-oriented immune pathways, facilitate angiogenesis and extracellular matrix remodeling, and play vital anti-inflammatory roles in wound repair. Currently, most TAMs within tumor stroma adopt the M2 phenotype, which promotes tumor initiation, disease progression, and eventual metastasis, thus serving as a prognostic marker in oncology (23, 24). TAM polarization is governed by multiple, dynamic signals from the TME. Classical M1 polarization is driven by factors such as lipopolysaccharide (LPS) and interferon-γ, which increase interleukin-2 (IL-2) while reducing IL-10 (21, 22). Conversely, M2 TAMs are polarized when exposed to IL-10, IL-4, and IL-13, displaying elevated IL-10 and reduced IL-12 levels (25).

## 3 The role of TAMs in CC

### 3.1 TAM-mediated immune suppression in CC

TME is predominantly composed of immune cells, including T lymphocytes, neutrophils, natural killer (NK) cells, macrophages, dendritic cells, and myeloid-derived suppressor cells (MDSCs) (26–29). As the predominant cellular component within the TME, TAMs foster immunosuppression in CC by releasing chemokines and cytokines that compromise the local immune response (30). Chemokines produced by TAMs, such as CCL5, CCL22, and CCL20, recruit regulatory T cells (Tregs), while cytokines including IL-10 and TGF-β promote Treg differentiation, thereby driving tumorigenesis. Furthermore, TAMs suppress antitumor functions of T cells and NK cells (19, 31), and cooperate with MDSCs, tumor-associated dendritic cells, and neutrophils to intensify immunosuppression (32). These macrophages also reduce T cell activity through enzymes including nitric oxide synthase (NOS) and arginase (ARGI) (33). In addition, they display high expression of ligands for PD-1 and CTLA-4 (for example, PDL1 and B7-H1), thereby weakening the cytotoxic capacity of T cells, NKT cells, and NK cells (34–36), which further hampers the host’s ability to eliminate colorectal cancer cells. Elevated CSF-1R levels on TAMs can facilitate immune evasion via Treg recruitment, potentially accelerating colon adenocarcinoma progression (37).

Siglec9 has been recognized as a crucial immune checkpoint molecule expressed on TAMs, significantly contributing to the development of an immunosuppressive TME (38–40). Studies indicate that Siglec9^+^ TAMs drive immune evasion in CC by facilitating suppressive cell infiltration and compromising CD8^+^ T-cell function (41). Leukemia inhibitory factor (LIF), a pleiotropic cytokine, is robustly produced in tumor-associated macrophages, and its administration endows them with potent immunosuppressive traits. MSC-1 interferes with LIF signaling by engaging epitopes overlapping the gp130 receptor binding region on LIF, prompting TAMs to adopt antitumor, proinflammatory attributes in a syngeneic CC mouse model (42). Additionally, heightened metastasis-associated protein 1 (MTA1) expression in CC fosters an immunosuppressive milieu exhibiting abundant CD8^+^ T cells but lacking classical macrophages (43). Moreover, eight pivotal enzymes governing amino acid metabolism in colon TAMs-ACADM, ACADS, GPX4, GSR, HADH, HMGCL, HMGCS1, and IDH1-trigger pyroptotic cell death in these macrophages, thereby potentially bolstering immune evasion in CC (44).

In addition to secreting cytokines and chemokines, TAMs mediate immune suppression through diverse receptor-ligand interactions and intracellular signaling cascades that affect T cells, NK cells, dendritic cells, and B cells. For instance, Tim-4^+^ macrophages in tumor cavities suppress CD8^+^ T cell anti-tumor responses, inhibiting cytotoxic immunity (45). Furthermore, TAM-derived PGE2 and TGF-β, along with various chemokines, disrupt dendritic cell (DC) maturation. This disruption destabilizes the equilibrium between innate and adaptive immune responses, while concurrently impairing the effector functions of both T cells and NK cells (46). Indoleamine 2,3-dioxygenase (IDO)-expressing TAMs metabolize tryptophan to kynurenine, an aryl hydrocarbon receptor (AHR) ligand that drives Treg differentiation while suppressing Th17 development and T-cell proliferation (47–50). As a consequence, TAMs establish an immune-privileged niche that enables CC cells to escape immunosurveillance and thrive within the TME.

### 3.2 TAM promotes angiogenesis

The vascular network sustaining tumor cells is pivotal in tumorigenesis and metastatic dissemination, making angiogenesis a critical process in tumor progression. Earlier investigations posited that this neovascularization was driven exclusively by tumor cells (51). However, emerging evidence shows that TAMs are major contributors to angiogenic regulation. Analyses of human CC tissues revealed that central angiogenic mediators-including S100A4, SPP1, and SPARC-are largely produced in stromal regions, particularly by TAMs, and strongly correlate with macrophage infiltration levels (52). Through immunohistochemical staining and image-based morphometry, Badawi et al. (53) examined microvascular density and macrophage presence in CC biopsy specimens and 15 benign adenomatous polyps, finding a marked link between elevated macrophage numbers and increased microvessel density in malignant tumors. Moreover, Luput et al. (54) demonstrated in an animal model that co-culturing C26 murine CC cells with TAMs significantly heightened total angiogenic protein levels in cell lysates. They also observed an inverse relationship between NF-κB activity and VEGF expression in C26 cells, indicating that TAMs boost angiogenesis by downregulating NF-κB. Additionally, CXCL1 is markedly upregulated in colorectal tumors and promotes neovascularization (55), while VEGF released by Siglec9^+^ TAMs further drives angiogenesis in CC (41).

### 3.3 TAMs involved in metastasis

Over 90% of cancer-related deaths are attributed to metastasis (56, 57). TAMs play essential roles in this process, contributing to epithelial-mesenchymal transition (EMT), local invasion, intravasation, systemic dissemination, extravasation, and final colonization that results in metastatic outgrowth (58–60). Within CC, TAMs facilitate metastatic progression by secreting various chemokines, proinflammatory molecules, and growth factors (61, 62). Jinushi and colleagues (63) reported that metastatic spread largely originates from tumor cells responding to cytokines released by TAMs, thus guiding migratory behavior. Furthermore, Xu et al. (64) observed that elevated Six1 protein levels in a murine colorectal cancer model foster TAM accumulation, which in turn amplifies metastatic potential. Moreover, TAMs increase EMR1/ADGRE1 expression in CC cells, potentially driving lymph node metastasis and disease progression through JAK2/STAT1,3 signaling (65). In colon adenocarcinoma (COAD), heightened CCL3 levels in both TAMs and tumor cells, acting via the CCL3-CCR5 axis, boost Akt-mediated migration and invasiveness (66). Additionally, CXCL1 fosters CC cell proliferation, enhances angiogenesis, and promotes M2 TAM recruitment through NF-κB/P300, ultimately advancing CC progression (55). The compound phenylhydrazine and pyrrolidinone sulfate 1 (PHPS1), identified as a potential phosphotyrosine inhibitor, can modulate M2-polarized TAMs and exosome secretion through the PI3K–AKT axis by downregulating SHP-2, thereby bolstering CC cell migration and invasion (67). Meanwhile, TAM-derived TGF-β1 instigates motility, invasiveness, and EMT in CC cells. Besides, the progression of EMT is facilitated by TAMs through activation of the CCL2/AKT/β-catenin signaling pathway (68). IL-6 secreted by TAMs interacts with the receptor complex involving glycoprotein 130 (gp130), thereby activating the JAK/STAT3 pathway in CC cells. This signaling cascade promotes EMT and chemoresistance (69, 70). Through heightened RBP-Jκ expression, CC cells release CXCL11, which elevates TGF-β1 levels in TAMs, further driving metastatic spread (71). Moreover, CCL14 fortifies M1 macrophage–mediated inhibition of CC cell proliferation and invasion, while negating the tumor-promoting influence of M2 macrophages (72).

### 3.4 TAM promotes chemoresistance

Macrophages, often referred to as TAMs, can hinder the success of chemotherapy. Numerous *in vivo* and *in vitro* investigations have shown their tumor-protective influence under various chemotherapeutic conditions (73, 74). Following chemotherapy, TAMs stimulate tumor advancement through a range of mechanisms, including increased recruitment of immunosuppressive macrophages, pro-tumor polarization, a Th17 response that favors malignancy, and heightened anti-apoptotic signaling in cancer cells (74). In particular, M2 macrophages are instrumental in driving tumor progression by sustaining an immunosuppressive local milieu (62). Moreover, a fraction of tumor cells known as cancer stem cells (CSCs) holds self-renewal potential and can produce malignant offspring. Due to their notable resilience to chemotherapy, CSCs serve as critical contributors to tumor recurrence (75–77). TAMs influence both CSC self-renewal and therapy resistance by controlling an intricate network of cytokines, chemokines, growth factors, and extracellular matrix molecules. Research indicates that TAMs induce chemoresistance by providing survival cues or activating anti-apoptotic pathways within malignant cells (62). This process often relies on macrophage-derived soluble mediators, although it may also involve extracellular matrix remodeling or direct cellular contacts (61). Inflammatory factors significantly influence the efficacy of therapies (78–81). Chemoresistance in CC cells frequently relies on the activation of STAT3, often driven by macrophage-derived IL-6 or other factors produced by macrophages (82). Moreover, FJX1 expression is positively associated with TAM infiltration and immune-related genes, such as TGFB1 and IL-10, as well as with immune-suppressive pathways involving TGFB1 and WNT1. By contrast, FJX1 demonstrates an inverse correlation with CD8^+^ T-cell abundance. Elevated FJX1 also diminishes immunotherapy efficacy while exacerbating therapy resistance (83). In parallel, hypoxia prompts HIF2α-dependent upregulation of dihydropyrimidine dehydrogenase in TAMs, thereby driving 5-FU resistance in CC (84). Studies have demonstrated a correlation between EMT and the development of resistance to chemotherapy (85). TAMs have been found to facilitate this transition through multiple mechanisms, including IL-8 (86), CCL20 (87), and the toll-like receptor 4/interleukin-10 (TLR4/IL-10) signaling cascade (88).

**Figure 1:**
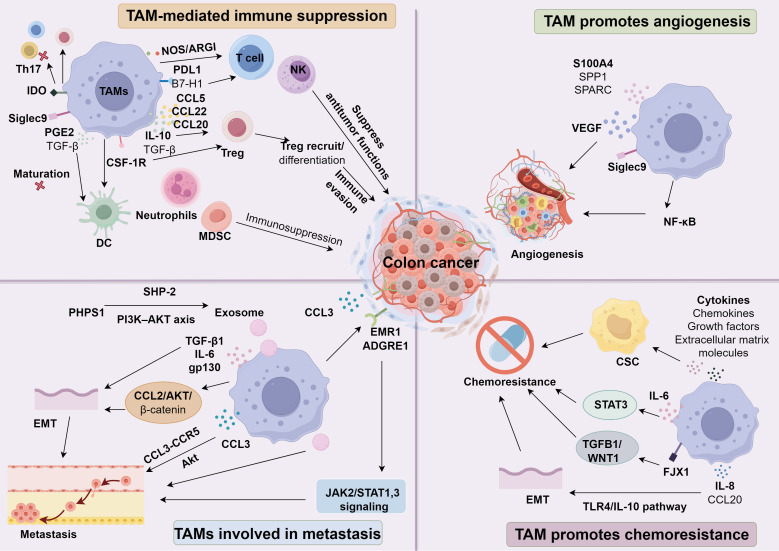
The role of TAMs in colon cancer.

Mounting evidence suggests that TAMs foster oncogenesis, and their increased numbers correlate with greater malignancy and worse patient outcomes across various cancers. Recent investigations underscore how TAMs remodel the TME. In CC, they primarily stimulate tumor progression by promoting angiogenesis, immune evasion, metastatic spread, and chemoresistance-partly through expanding cancer stem cell populations. During immunotherapeutic interventions, TAMs can also reshape the microenvironment to enable anti-tumor responses. Accordingly, emerging clinical data indicate that therapeutic strategies directed at TAMs-such as limiting their infiltration, altering M2/M1 polarization, or inhibiting key signaling pathways linked to TAM-associated antigen functions-may provide a promising route to enhance tumor immunotherapy (**Figure 1**).

## 4 Therapies targeting TAMs

### 4.1 Reduction of TAMs

Elevated levels of TAMs in tumors are strongly correlated with the progression of colorectal cancer (89). Current therapeutic strategies aim to restrict the infiltration of macrophage precursors and eliminate existing TAM populations within the TME. This process is facilitated by several chemokines, including CCL3, CCL4, CCL5, CCL22, and CXCL8, which are highly expressed in various tumors and promote TAM migration (90–92). Additionally, cytokines such as CSF-1 and EMAPII (93, 94), as well as growth factors like VEGF, endothelin-2, and PDGF, further enhance the recruitment of monocytes and macrophages (95–98). In hypoxic regions of tumors, HIF-1α plays a critical role in reprogramming TAMs toward a protumor phenotype by altering gene expression (99, 100). HIF-1α upregulates CXCR4 and induces CXCL12, which drives the recruitment of monocytes and macrophages into these oxygen-deprived areas (101, 102). It also increases VEGF-A expression in TAMs, creating a self-sustaining cycle that reinforces their survival and persistence (103). Meanwhile, hypoxia triggers MKP-1, which curbs ERK1/2 and p38 MAPK activity, restricting TAM mobility and causing them to remain under hypoxic stress (104). Additionally, hypoxia downregulates CCR2 and CCR5 on macrophages, reducing their chemotactic responsiveness (105). Targeting TAMs has emerged as a promising strategy to combat cancer progression. For instance, in an orthotopic CC xenograft mouse model, the CSF-1R inhibitor PLX3397 effectively reduced M2 macrophage populations, and its combination with anti-PD-1 therapy significantly suppressed tumor growth (106). Furthermore, FAP-modified, tumor-derived exosome-like vesicles (eNVs-FAP) demonstrated potent anticancer vaccine activity by inducing robust tumor-specific cytotoxic T lymphocyte (CTL) responses. This approach also reduced immunosuppressive components, including M2-TAMs, in the CC model (107).

### 4.2 Modulation of TAM polarization

In CC, TAMs typically adopt an M2-oriented phenotype, contrasting with M1 macrophages, which display a classically activated profile. These cells actively collaborate with tumor elements, primarily via the TME driving them toward an immunosuppressive M2 state that promotes vascularization, tumor expansion, invasion, and metastatic spread (36). Notably, M1 and M2 macrophages can switch between phenotypes under changing conditions. According to Georgoudaki et al. (108), immune checkpoint treatments in a mouse MC38 colorectal cancer model strengthened antitumor defenses by shifting TAMs toward an M1 phenotype and elevating tumor immunogenicity. Stimulating the STING pathway within TAMs presents a potential approach for managing CC. Recent data indicate that the prodrug STING agonist GB2 redirects TAM polarization by targeting TREM2 within malignant tissue, thereby enhancing CC immunotherapy (109). Moreover, inhibiting phosphoglycerate mutase 1 can diminish M2 polarization in CC cells, thereby restricting TAM-driven disease progression (110). Blue light exposure was further shown to impede M2 polarization in TAMs during CC (111). Curcumin mitigates malignant features of CC cells by controlling TAM M2 polarization and metastasis-associated colon cancer 1 (MACC1) levels (112). Through dual targeting of M2-oriented TAMs and TGF-β1-dependent positive feedback mechanisms, paclitaxel hinders CC advancement (113). Tasquinimod modulates myeloid cell recruitment in CC, inducing a shift from immunosuppressive M2-like TAMs with pro-angiogenic traits toward M1-like macrophages, consistent with its documented effects on vascularization, immune evasion, and metastatic activity (114). Within peptide-guided cancer therapy, recent investigations examined IFN-γ liposomes engineered with an M2-targeting peptide, selectively adhering to anti-inflammatory TAMs. Findings revealed improved uptake and antitumor activity of liposomal IFN-γ in C26 CC mouse models (115). Mesobuthus eupeus venom also shows promise against tumor expansion and metastasis by redirecting macrophages from M2 to M1 polarity (116). Furthermore, inhibiting CD39 with polyoxotungstate (POM-1) curbed CC tumor burden in mice and was associated with an upsurge of M1-like TAMs in tumor tissue (117).

### 4.3 Novel and ongoing clinical trials targeting TAMs

In CT26 CC models, HDAC6 inhibitor AVS100 increased pro-inflammatory tumor-infiltrating macrophages and CD8 effector T cells with inflammatory gene profiles (118). Recently, AVS100’s favorable safety profile led to FDA IND approval, enabling clinical trials (118). A Phase I trial of Trebananib (Angiopoietin 1/2 inhibitor) with Pembrolizumab in CC showed reduced T cells and increased immunosuppressive macrophages post-resistance (119). Zhang et al. identified CD155 expressing on M2 type TAMs as therapeutic target in digestive cancers, with CD155-directed CAR-T cells showing promise in clinical evaluations, including CC (120). These findings underscore the translational potential of targeting TAMs to enhance immunotherapy (**Table 1**).

**Table 1:** Potential therapeutic approaches targeting TAMs in colon cancer.

Strategy	Treatments	Mechanism
Depletion of TAMs	CSF-1R inhibitors (e.g., PLX3397)	Block recruitment and survival of TAMs by inhibiting CSF-1/CSF-1R signaling
	eNVs-FAP vaccine	Induce robust tumor-specific T-cell responses, reducing M2 TAMs
Inhibition of TAM Recruitment	Targeting chemokines (CCL2, CCL3, CXCL8/12)	Interfere with chemokine-driven monocyte infiltration
	Blocking hypoxia-induced pathways (HIF-1α)	Suppress hypoxia-driven TAM recruitment and retention
Reprogramming TAMs	STING agonists (e.g., GB2)	Activate pro-inflammatory M1 phenotype
	Allosteric inhibitors (e.g., phosphoglycerate mutase 1 inhibitors)	Downregulate immunosuppressive signaling
Modulating the TME or Checkpoints	Anti-PD-1/PD-L1 antibodies; Anti-CTLA-4 antibodies	Reduce immunosuppressive signals from TAMs and other TME components; Restore cytotoxic T-cell function
Novel and Ongoing Clinical Trials	HDAC6 inhibitor AVS100; Trebananib (Angiopoietin 1/2 inhibitor) with Pembrolizumab; CD155-directed CAR-T	Increase tumor-infiltrating macrophages and CD8 effector T cells; By identifying the M2 immunosuppressive marker CD155, showing promise in models of gastrointestinal tumors, including CC

## 5 Conclusion

TAMs are central to colon cancer progression by fostering immune evasion, neovascularization, metastasis, and resistance to chemotherapy. The predominance of M2-oriented TAMs correlates with unfavorable clinical outcomes. Efforts to minimize their infiltration, alter their polarization, or disrupt their immunosuppressive traits may greatly enhance the efficacy of established colon cancer interventions. Approaches that focus on TAMs-used individually or in conjunction with existing immunotherapies-could form promising, patient-specific strategies. While obstacles remain in effectively neutralizing TAMs, continued investigations reveal new therapeutic prospects that hold potential for significantly influencing colon cancer management. Future directions for TAM research should include (1) leveraging advanced molecular and imaging techniques (e.g., single-cell RNA sequencing, multiplex immunofluorescence) to dissect TAM heterogeneity within the tumor microenvironment; (2) exploring combinatorial therapeutic strategies that integrate TAM modulation (via polarization reprogramming or depletion) with immune checkpoint inhibitors or targeted therapies; (3) identifying novel biomarkers that can accurately assess TAM activity and predict patient response to treatment; and (4) elucidating the role of the gut microbiome and other systemic factors in regulating TAM function. By pursuing these multidisciplinary approaches, researchers may uncover innovative interventions that enhance antitumor immunity and substantially improve clinical outcomes in CC.
